# Hypertension and COVID-19: Ongoing Controversies

**DOI:** 10.3389/fcvm.2021.639222

**Published:** 2021-02-17

**Authors:** Marijana Tadic, Sahrai Saeed, Guido Grassi, Stefano Taddei, Giuseppe Mancia, Cesare Cuspidi

**Affiliations:** ^1^Department of Cardiology, University Hospital “Dr. Dragisa Misovic - Dedinje”, Belgrade, Serbia; ^2^Department of Heart Disease, Haukeland University Hospital, Bergen, Norway; ^3^Department of Cardiology, University of Milan-Bicocca, Milan, Italy; ^4^Department of Clinical and Experimental Medicine, University of Pisa, Pisa, Italy; ^5^University of Milano-Bicocca, Milano and Policlinico di Monza, Monza, Italy; ^6^Department of Cardiology, Istituto Auxologico Italiano, Scientific Institute for Research, Hospitalization and Healthcare, Milan, Italy

**Keywords:** hypertension, COVID - 19, blood pressure, antihypertensive therapy, comorbidities

## Abstract

Coronavirus disease 2019 (COVID-19) has become a worldwide pandemic responsible for millions of deaths around the world. Hypertension has been identified as one of the most common comorbidities and risk factors for severity and adverse outcome in these patients. Recent investigations have raised the question whether hypertension represents a predictor of outcome in COVID-19 patients independently of other common comorbidities such as diabetes, obesity, other cardiovascular diseases, chronic kidney, liver, and pulmonary diseases. However, the impact of chronic and newly diagnosed hypertension in COVID-19 patients has been insufficiently investigated. The same is true for the relationship between blood pressure levels and outcomes in COVID-19 patients. It seems that the long discussion about the impact of angiotensin-converting enzyme inhibitors (ACEI) and blockers of angiotensin I receptors (ARB) on severity and outcome in COVID-19 is approaching an end because the large number of original studies and meta-analyses discarded the initial findings about higher prevalence of ACEI/ARB use in patients with unfavorable outcomes. Nevertheless, there are many controversies in the relationship between hypertension and COVID-19. The aim of this review article is to provide a clinical overview of the currently available evidence regarding the predictive value of hypertension, the effect of blood pressure levels, the impact of previously known and newly diagnosed hypertension, and the effect of antihypertensive therapy on the severity and outcomes in COVID-19 patients.

## Introduction

Coronavirus disease 2019 (COVID-19), induced by Severe Acute Respiratory Syndrome Coronavirus 2 (SARS-CoV-2), has become a worldwide pandemic that is responsible for millions of deaths around the world. Hypertension, diabetes, and cardiovascular diseases were soon identified as common comorbidities in COVID-19 patients (1, 2). The following studies revealed that hypertension is an important risk factor for adverse outcomes in COVID-19 patients (3, 4). Initial studies reported hypertension as an independent predictor of hospitalization, an advanced stage of pneumonia, admission to the intensive care unit (ICU), and mortality in these patients (3–5). Later investigations raised the question whether hypertension would be a predictor of outcome in COVID-19 patients independently of diabetes, obesity, and other cardiovascular diseases (6, 7). Furthermore, the majority of studies did not make any distinction between patients with chronic and new-onset of hypertension in COVID-19 patients, which could significantly impact final results. The relationship between blood pressure level and susceptibility to SARS-CoV-2 or outcome in COVID-19 patients has been insufficiently investigated, and potential blood pressure target value in these patients is still unknown.

There was a long discussion regarding the impact of antihypertensive therapy in COVID-19 patients and particularly of angiotensin-converting enzyme inhibitors (ACEI) and blockers of angiotensin I receptors (ARB). After initial reports that showed higher prevalence of use of these medications in COVID-19 patients with cardiac injury and more severe course of disease (8, 9), numerous original studies and meta-analysis reported no relationship with severity or mortality in COVID-19 patients (10, 11) or even benefit of taking renin-angiotensin-aldosterone inhibitors in COVID-19 patients (12, 13).

The aim of this review article is to provide an overview of the current evidence on controversies regarding hypertension in COVID-19 patients: predictive value of hypertension, effect of blood pressure (BP) level and control, influence of new-onset hypertension and impact of antihypertensive therapy.

## Is Hypertension an Independent Predictor of Outcome in COVID-19 Patients?

Initial studies were focused on prevalence of different comorbidities, including the impact of various risk factors on susceptibility, severity and mortality of COVID-19 (3–5). Later investigations revealed association between hypertension and more advanced stages of disease and mortality (14, 15). However, majority of them did not include diabetes and obesity in multivariable analysis.

A recent study demonstrated that hypertension alone was not an independent predictor of outcome, but only in combination with diabetes or some other risk factor (6). One should also notice that some researches did not show any impact of neither hypertension nor diabetes on outcome in COVID-19 patients (16), whereas other investigations reported that both hypertension and diabetes with or without obesity were independently associated with adverse outcome (3).

Bauer et al. suggested that hypertension was independent predictor of severe form of COVID-19 only in patients younger than 65 years, but not in the whole study population (17). On the other hand, diabetes and congestive heart failure were independent predictors both in patients younger than 65 years and in all participants. Barrera et al. included 15,794 participants and reported that hypertension and diabetes separately were significant predictors of ICU and mortality, but not with severe COVID-19 (18). Interestingly, concomitant presence of hypertension and diabetes was not predictor of severe COVID-19 (18).

An investigation that included almost 4,000 critically ill COVID-19 patients that were hospitalized in ICU showed that hypertension, diabetes, cardiovascular diseases, hypercholesterolemia, chronic kidney disease, and other comorbidities were predictors of mortality in these patients (19). However, among these comorbidities only diabetes and hypercholesterolemia were independent predictors (19). A study that involved only hypertensive patients reported that diabetes was not an independent prognostic factor, whereas age and chronic kidney disease were independent predictors. The same study demonstrated that hypertension, diabetes and obesity were independent predictors of severe COVID-19 in both sexes and obesity was stronger predictor in patients younger than 50 years, whereas the interaction between hypertension and diabetes with age was not noticed (20). However, the authors did not perform adjustment for all comorbidities like in the first mentioned from the same cohort of patients.

Furthermore, the National Cohort Study in England investigated 19,256 COVID-19–related ICU admissions and revealed that patients with type 2 diabetes were at increased risk of mortality independently of hypertension, chronic respiratory disease, chronic heart disease, chronic renal disease, chronic liver disease and other potential risk factors (21). Nevertheless, the recent investigation showed no association between hypertension and mortality or acute respiratory distress syndrome (ARDS) in COVID-19 patients (6). The authors reported that hypertension only together with diabetes was independent predictor of mortality and ARDS in COVID-19 patients (6). Moreover, diabetes alone was also independently related with adverse outcomes in these patients. On the other hand, Gupta et al. revealed that only body mass index ≥ 40 kg/m^2^ and coronary artery disease were independent predictors of 28-day mortality in COVID-19 patients (16). Hypertension, diabetes, heart failure and chronic pulmonary obstructive disease were not independently associated with lethal outcome in these patients. Table 1 summarizes findings from described studies.

**Table 1:** Hypertension as independent predictor of outcome in COVID-19.

**Reference**	**Sample size**	**Age**	**Women (%)**	**Hypertension (%)**	**Other important findings**
Sun et al. (6)	3,400	61 (50–68)	1,751 (51)	1,782 (52)	Hypertension only together with diabetes was independent predictor of mortality and ARDS.
Bauer et al. (17)	1,449	54.7 ± 22.5	920 (63)	525 (36)	Hypertension was independent predictor of severe form of COVID-19 only in patients younger than 65 years, but not in the whole study population.
Barrera et al. (18)	15,794	–	–	–	Hypertension and diabetes separately were significant predictors of ICU and mortality, but not with severe COVID-19.
Grasselli et al. (19)	3,988	63 (56–69)	800 (20)	1,643 (41)	Diabetes and hypercholesterolemia, but not hypertension, were independent predictors of mortality in COVID-19 patients hospitalized in ICU.
Dennis et al. (21)	19,256	66	7,683 (40)	5,657 (29)	Patients with type 2 diabetes were at increased risk of mortality independently of hypertension, chronic respiratory disease, chronic heart disease, chronic renal disease, chronic liver disease and other potential risk factors.
Gupta et al. (16)	2,215	60.5 ± 14.5	779 (35)	1,322 (60)	Body mass index ≥ 40 kg/m^2^ and coronary artery disease, but not hypertension and diabetes, were independent predictors of 28-day mortality in COVID-19 patients.

*ARDS, acute respiratory distress syndrome; ICU, intensive care unit*.

There are several major limitations of mentioned studies: retrospective nature, confounding factors that were not measured, lack of information regarding duration of hypertension, diabetes and other comorbidities, as well as missing or incomplete data about antihypertensive and anti-diabetic therapy.

## The Influence of Blood Pressure Control in COVID-19

Data regarding the impact of BP level on susceptibility, severity or outcome of COVID-19 patients are scarce. Majority of studies and particularly those published at the beginning of pandemic were based on anamnestic data and therefore were not fully reliable. Recently published studies investigated the impact of BP control on outcome in COVID-19 patients and provided more detailed insight (22, 23).

Ran et al. investigated 803 hypertensive patients with COVID-19 and found that average systolic BP was independent predictor of only heart failure development in these COVID-19 patients (22). After adjustment for confounding factors (systolic and diastolic BP on admission, age, sex, smoking, alcohol consumption, and comorbidities (cancer, diabetes, coronary heart disease, cerebrovascular disease, COPD, chronic liver disease, and chronic kidney disease), the remaining significant predictors for heart failure were average systolic BP and pulse pressure, and an increase in systolic BP variability was also marginally associated with an increased hazard of heart failure. Increased BP variability was significantly associated with higher risks of mortality and ICU admission, respectively. The authors showed that the risks of COVID-induced heart failure development significantly increased in patients with high systolic BP, but this trend was less evident for diastolic BP (22). This finding implies that high BP is an important predictor of adverse outcome and suggests that systolic BP should be the primary target of BP control in COVID-19 patients. However, high BP variability was related with high risks of mortality and ICU admissions, underlying the importance of maintenance of stable in-hospital BP in these patients. Increased BP variability could reflect increased arterial stiffness and endothelial dysfunction that are associated with cardiovascular events (24, 25). Additional explanation could be a sudden BP decline because of progressive deterioration of underlying conditions.

Chen et al. reported gradual increase in lethal outcome, septic shock, ARDS, respiratory failure, mechanical ventilation and ICU admission from normotensive patients with COVID-19, throughout patients with grade I hypertension, to those with grade II and III hypertension (23). Even though trend existed for all outcomes, one must admit that the significant difference was not noticed between normotensive patients and participants with grade I hypertension, as well as between grade II and III hypertension. Interestingly, the length of disease and symptoms gradually increased with grade of hypertension. In multivariable analysis hypertension grade ≥2 was independently associated with adverse events. However, diabetes was not included in multivariable analysis despite its significant proportion among COVID-19 patients in total study population and particularly in hypertensive participants (23).

Determination of the relationship between BP and COVID-19 outcome is not an easy task due to its high variability and dependency on comorbidities. Furthermore, both studies investigated hospitalized patients, which means that BP was measured after admission and not after symptoms onset and therefore COVID-19 could already influence BP.

## Chronic vs. New-Onset Hypertension in COVID-19

One of the main challenges in assessment of the relationship between hypertension and COVID-19 is the absence of data regarding the ratio of patients with hypertension before hospital admission. Namely, patients with chronic hypertension have significant endothelial dysfunction, which is crucial in the pathogenesis of cardiovascular complications in COVID-19 (25). Chronically hypertensive patients often have target organ damage that increases susceptibility for SARS-CoV-2 and elevates the risk of unfavorable outcomes in COVID-19 patients.

Data regarding the impact of known and newly diagnosed hypertension in COVID-19 patients are very limited. Ran et al. found that poor BP control was independently associated with adverse outcomes in COVID-19 patients with chronic hypertension (22). Chen et al. reported that stage I chronic hypertension was present in only 37% of hospitalized COVID-19 patients, whereas the prevalence of chronic hypertension stages II and III was significantly higher (61 and 70%, respectively) (23). This shows that newly diagnosed hypertension was present in a significant portion of COVID-19 patients. The investigators demonstrated that unfavorable outcomes (mortality, septic shock, respiratory failure, ARDS, ICU admission) gradually increased with BP elevation (23). However, the authors did not make separate analyses for patients with known and newly diagnosed hypertension.

Xiong et al. showed that almost 40% of patients with known hypertension did not receive any antihypertensive medication (26). Nevertheless, the occurrence of adverse events did not differ between patients who were previously treated with anti-hypertensive medications and those who did not receive therapy despite hypertension. A significant limitation is the small number of hypertensive patients in this study (*n* = 71), which is not enough to make a conclusion (26). One should also keep in mind that the majority of studies regarding COVID-19 have come from China, where traditional medicines are frequently used instead of formal medications, including antihypertensive drugs.

Data regarding the relationship between known and newly diagnosed diabetes among COVID-19 patients potentially might be used as the model for the association between chronic and newly diagnosed hypertension with outcome in these patients. It is already well-established that diabetes is associated with elevated risk of adverse outcomes in COVID-19 patients. Nevertheless, one should notice that COVID-19 could induce diabetes with its metabolic complications and insulin therapy requirement. Li et al. found that newly diagnosed diabetes was associated with higher mortality than known diabetes in hospitalized COVID-19 patients (27). Similar results were reported from the Italian group (28). Higher glucose levels at admission were related to COVID-19 severity, with a stronger association among patients without as compared to those with known diabetes.

It is evident that this topic deserves further investigation because whether newly diagnosed hypertension potentially has a more negative effect than chronic hypertension should be explained, in the same way as the comparison of newly diagnosed diabetes with known diabetes. This would have significant clinical and particularly therapeutic implications in COVID-19 patients.

## Antihypertensive Therapy in COVID-19

SARS-CoV-2 enters human host cells upon binding to angiotensin-converting enzyme 2 (ACE2)—a molecule functioning both as the main trans-membrane receptor for the virus and a component of the renin-angiotensin system —the key BP regulating cascade. Because renin-angiotensin-system inhibitors increase ACE2 levels, the potential negative effect of ACEI or ARB has been largely discussed since the beginning of COVID-19 pandemic. This hypothesis was supported by initial findings that these medications were more frequently used in COVID-19 patients with cardiac injury or in those with severe form of disease (8, 9). Nevertheless, later reports failed to show any negative relationship between adverse outcome and use of ACEI and ARB in COVID-19 patients (10–13).

A large study from the United Kingdom that included 16,866 patients with COVID-19 events and 70,137 matched controls showed that ACEIs and ARBs were associated with lower risk of COVID-19 diagnosis (28). In fully adjusted analyses, calcium channel blockers and thiazide diuretics were also associated with lower risk of COVID-19. Interestingly, beta-blockers were initially associated with increased risk, but this relationship disappeared in a multivariable-adjusted model (28). In adjusted analyses, patients treated with ACEIs or ARBs had similar mortality to patients treated with beta-blockers, calcium channel antagonists, and other antihypertensive medications or patients receiving no antihypertensive therapy (28).

A study that analyzed 880 COVID-19 patients from Germany and the Netherlands reported that use of ACEI/ARB and diuretics was not related to worse outcomes; instead, use of beta-blockers was associated with better outcomes, and use of calcium channel blockers with poorer outcomes (29). The model was adjusted only for age, sex, and diabetes and therefore not fully conclusive, if we consider the fact that many other confounding factors (comorbidities in the first place) were not included (29). There is a hypothesis that some beta-blockers, such as carvedilol, unlike ACEI and ARB, decrease the expression of ACE2 and suppress the properties of interleukin-6, which potentially could help in treatment of COVID-19 patients (30). However, this still remains in the domain of hypothesis.

Gao et al. reported no difference in mortality, time from onset of symptoms to discharge, COVID-19 severity, and percentage of ventilation between the cohort of patients who were treated with ACEI/ARB and those treated with beta-blockers, calcium channel blockers, and diuretics (31). Unfortunately, the authors did not investigate the influence of each antihypertensive class separately. Other Chinese study reported no association between any antihypertensive class (ACEI/ARB, beta-blockers, calcium channel blockers, and diuretics) and the composite endpoint, which was defined as admission to an ICU, need for mechanical ventilation, or a fatal outcome (26).

A Massachusetts community-based observational study showed that no antihypertensive medications were related to increased risk of severe COVID-19 (17). The authors investigated each of five antihypertensive classes separately. Similar results were reported in a large meta-analysis that included 2,100,587 participants (32). The investigators observed no association between prior usage of antihypertensive medications, including ACEIs/ARBs, calcium channel blockers, beta-blockers, or diuretics, and the risk or severity of COVID-19. Interestingly, when the analysis included only hypertensive patients, prior usage of ACEIs/ARBs was related to lower severity and mortality of COVID-19.

The large Italian population-based study that matched 6,272 COVID-19 patients and 30,759 subjects according to sex, age, and municipality of residence, showed that therapy with ACEIs and ARBs was more prevalent in COVID-19 patients than among their counterparts because of higher prevalence of CV disease in COVID-19 patients (33). Nevertheless, there was no association between use of ACEIs or ARBs and the risk of COVID-19.

Considering the confusion about the use of ACEI/ARBs in COVID-19 patients with hypertension that appeared at the beginning of the pandemic, hypertension societies around the globe were forced to publish statements that should encourage the maintenance of ongoing antihypertensive therapy and the following of current guidelines (34), which include the use of ACEI/ARB and the avoidance of replacing or switching ACEI/ARB to another antihypertensive medication (35–37).

Large recently published studies and meta-analysis have significantly reduced initial uncertainties regarding the use of ACEI and ARB in treatment of COVID-19 patients. Available data indicate that all antihypertensive classes are safe in this group of patients. However, prospective studies with a large number of patients with accurate data regarding antihypertensive therapy before and during COVID-19 would be very much appreciated.

## Differences Among Countries

Data regarding incidence and mortality of COVID-19 significantly changed during pandemic and particularly between different countries. Sorci et al. used data on the temporal trajectory of the case fatality rate provided by the European Center for Disease Prevention and Control, as well as country-specific data (38). The authors reported that temporal trajectories of case fatality rate vary significantly among countries. The main factors associated with temporal changes were comorbidities, demographic, economic, and political parameters. Countries with the highest prevalence of cardiovascular, cancer, and chronic respiratory diseases showed the highest levels of COVID-19 CFR (37). However, these are still preliminary data because information from all countries is updated on a daily basis and final conclusions will be published once the pandemic is over.

## Future Directions

Many questions regarding the effects of hypertension, BP level, BP control, and antihypertensive therapy have been raised since the beginning of COVID-19 pandemic. A large number of studies have been published over a very short time period, which unfortunately does not guarantee their quality. Many questions remained without adequate answers. This is particularly true for the influence of BP levels and control on outcomes for COVID-19 patients. There is still not enough evidence about the effects of known and newly diagnosed hypertension on the severity and outcomes of COVID-19 for patients. A large number of studies considered the association of different antihypertensive classes of medications with the outcomes in these patients, but almost all of them are retrospective investigations or meta-analyses. It is evident that well-conducted research with a significant number of hypertensive patients is necessary to resolve current controversies in the relationship between hypertension and COVID-19.

## Author Contributions

MT: writing the article. SS: searching the literature and review. ST, GG, and GM: detailed review with constructive remarks that substantially changed the article. CC: conceptualization of the article and constructive review. All authors contributed to the article and approved the submitted version.

## Conflict of Interest

The authors declare that the research was conducted in the absence of any commercial or financial relationships that could be construed as a potential conflict of interest.
